# Low Power Consumption Substrate-Emitting DFB Quantum Cascade Lasers

**DOI:** 10.1186/s11671-017-2281-8

**Published:** 2017-09-02

**Authors:** Chuan-Wei Liu, Jin-Chuan Zhang, Zhi-Wei Jia, Ning Zhuo, Shen-Qiang Zhai, Li-Jun Wang, Jun-Qi Liu, Shu-Man Liu, Feng-Qi Liu, Zhan-Guo Wang

**Affiliations:** 1 Key Laboratory of Semiconductor Materials Science, Institute of Semiconductors, Chinese Academy of Sciences, Beijing Key Laboratory of Low Dimensional Semiconductor Materials and Devices, Beijing, 100083 People’s Republic of China; 2 College of Materials Science and Opto-Electronic Technology, University of Chinese Academy of Sciences, Beijing, 101408 People’s Republic of China

**Keywords:** Quantum cascade laser, Substrate-emitting, Low power consumption

## Abstract

In the present work, an ultra-low power consumption substrate-emitting distributed feedback (DFB) quantum cascade laser (QCL) was developed. The continuous-wave (CW) threshold power dissipation is reduced to 0.43 W at 25 °C by shortening the cavity length to 0.5 mm and depositing high-reflectivity (HR) coating on both facets. As far as we know, this is the recorded threshold power dissipation of QCLs in the same conditions. Single-mode emission was achieved by employing a buried second-order grating. Mode-hop free emission can be observed within a wide temperature range from 15 to 105 °C in CW mode. The divergence angles are 22.5^o^ and 1.94^o^ in the ridge-width direction and cavity-length direction, respectively. The maximum optical power in CW operation was 2.4 mW at 25 °C, which is sufficient to spectroscopy applications.

## Background

In recent years, quantum cascade lasers (QCLs) have undergone a rapid development and become the most promising source in the mid-infrared frequency region [1–3]. Benefited from their high power, single-mode operation and compact size, distributed-feedback (DFB) QCLs have been widely used in many applications such as trace-gas sensing, free space communication, and substance analysis [4–6]. However, the remaining disadvantage of QCLs is their high electrical power dissipation, which has limited their application in some portable and highly integrated systems. To decrease the power dissipation, the most straightforward method is to decrease the geometry size of the device, such as shortening the cavity and narrowing the ridge. High-reflectivity (HR) coating is also very effective for reducing the mirror loss. Some studies have been done to reduce the threshold power dissipation of Fabry-Perot (FP) QCLs by using a short cavity and depositing HR coating [7] or partial high-reflectivity (PHR) coating on the facets [8], in which the dissipated power as low as 1.2 W at 22 °C and 0.83 W at 25 °C have been demonstrated by A. C. Richard et al. and Y. Bai et al., respectively. These methods could also be applied to DFB devices. In 2014, Ryan M. Briggs et al. reported a single-mode DFB QCL emitting at 4.8 μm with a CW threshold power consumption of 0.76 W and maximum optical power of about 17 mW at 20 °C [9]. In 2015, A. Bismuto et al. demonstrated short cavity, narrow ridge single-mode DFB QCLs emitting at 4.5 μm with CW threshold dissipated power as low as 0.5 W at 20 °C [10]. The maximum optical power is about 150 mW; however, the injected electrical power is more than 6 W. Other methods such as doping optimization and low-period active structure have also been investigated [7, 11]. For edge-emitting QCLs, HR coating is commonly deposited on the back facet and leaving the front facet uncoated or PHR coated to reduce mirror loss meanwhile maintaining the optical power emitted from the front facet. Instead, both facets can be HR coated for substrate-emitting to further decrease the mirror loss since the light is emitted from substrate instead of front facet. Besides, improved far-field distributions can be expected from substrate-emitting QCLs [12, 13]. According to our recent work, a substrate-emitting DFB QCL with low threshold power dissipation of 1.27 W at 20 °C was obtained by depositing HR coating on both facets [14]. The active region in Ref [14] consists of 40 superlattice periods, and the threshold voltage is about 13 V. A lower threshold voltage, and thus, lower threshold power dissipation can be expected if the period number of active region is decreased. The cavity length of 1 mm could also be further shortened by properly designing the buried second-order grating to decrease the threshold power dissipation.

In the present work, an ultra-low power consumption substrate-emitting DFB QCL was developed. The threshold power dissipation working in CW mode is as low as 0.4 W at 15 °C and 0.43 W at 25 °C by shortening the cavity length to 0.5 mm and depositing HR coating on both facets. The maximum optical power in CW mode is 2.4 mW at 25 °C, which is sufficient to spectroscopy applications. Single-mode emission was achieved by employing a buried second-order grating. The divergence angles are 22.5^o^ and 1.94^o^ full width at half maximum (FWHM) in the ridge-width direction and cavity-length direction, respectively. The double-lobed far-field distribution in cavity-length direction indicates that anti-symmetric mode is favored. These devices can operate in CW mode without mode-hop in a wide temperature range from 15 to 105 °C and will be very suitable in high-integrated portable applications.

## Methods

**Fig. 1:**
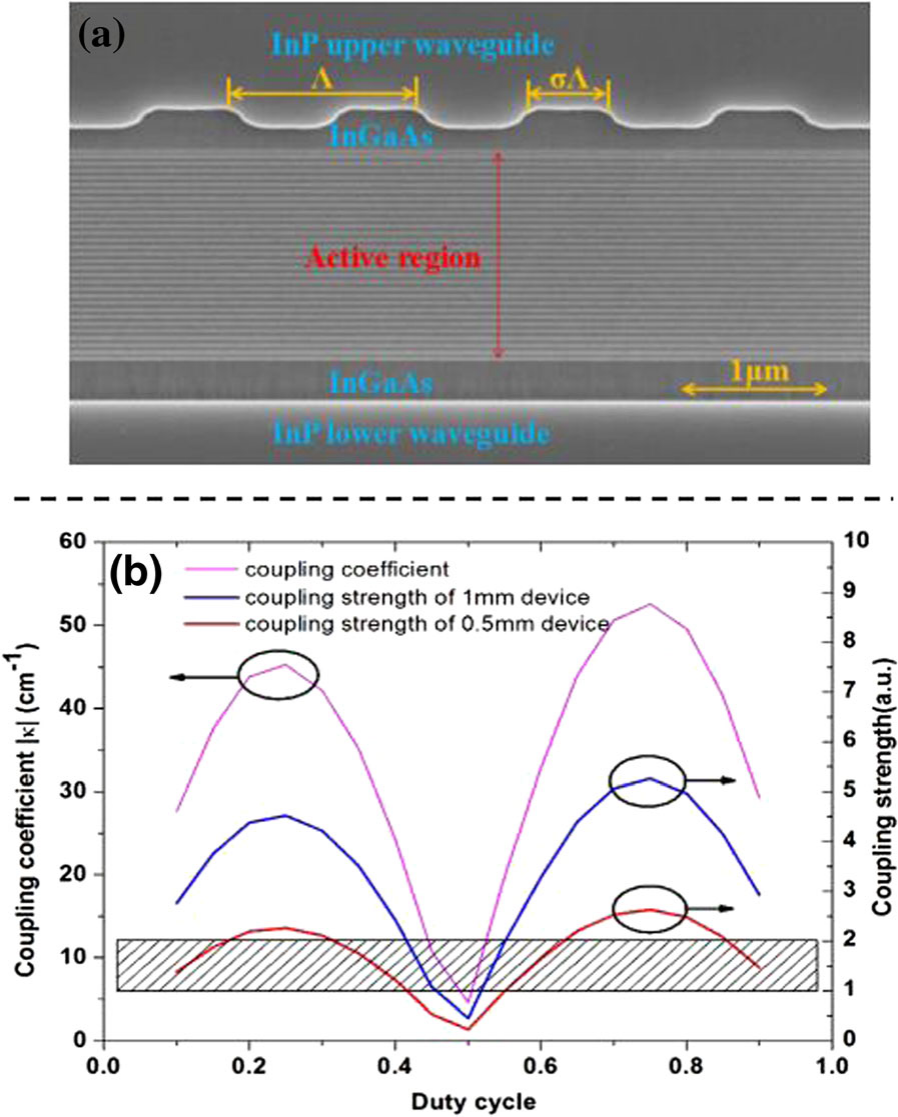
**a** The SEM image of the buried grating and **b** the simulated coupling coefficient and coupling strength of the buried second-order grating with COMSOL

The active region of the device was based on strain-compensated two-phonon resonant design emitting at ~ 4.6 μm. The wafer was grown on an n-doped (Si, 2 × 10^17^ cm^− 3^) InP substrate by solid-source molecular beam epitaxy (MBE). Thirty stages of In_0.67_Ga_0.33_As/In_0.36_Al_0.64_As quantum wells and barriers were included in the active core, which was similar to Ref. [15] The entire layer sequence was as follows: 1.2-μm-thick lower cladding layer (Si, 2.2 × 10^16^ cm^− 3^), 0.3-μm-thick n-In_0.53_Ga_0.47_As layer (Si, 4 × 10^16^ cm^− 3^), 30 active/injector stages, 0.3-μm-thick n-In_0.53_Ga_0.47_As layer (Si, 4 × 10^16^ cm^− 3^), and top waveguide cladding player. To fabricate the buried grating, the top waveguide cladding layer was removed down to the upper InGaAs layer. The second-order grating with a period of *Λ* = 1.42 μm (duty cycle *σ* = 0.45, depth *d* = 130 nm) was defined on the 0.3-μm-thick upper InGaAs layer by holographic lithography technology and wet chemical etching. Figure 1a shows the scanning electron microscope (SEM) image of the buried second-order grating. After that, a 3-μm-thick low-doped InP layer (Si, 2.2 × 10^16^ cm^− 3^) followed by a 0.15 μm gradually doped InP layer (Si, from 1 × 10^17^ to 3 × 10^17^ cm^− 3^) and a 0.4-μm high-doped InP cladding layer (Si, 5 × 10^18^ cm^− 3^) were accomplished in sequence as upper cladding by metal-organic vapor phase epitaxy (MOVPE).

Following the implementation of the regrowth, the wafer was etched into double-channel waveguide structure with an average core width of 7 μm. Then, a 450-nm-thick SiO_2_ was deposited by plasma-enhanced chemical vapor deposition (PECVD) for insulation. A 2-μm-wide electron injection window was patterned on SiO_2_ layer of the ridge, and electrical contact was provided by a Ti/Au layer. For better heat dissipation, an additional 4-μm-thick Au layer was electroplated. Before actually cleaving, massive simulations for the design of the second-order buried grating was implemented with finite element method software (COMSOL), which was similar to Ref. [16] After thinning to 150 μm, the waveguide was cleaved to 0.5- and 1-mm-long devices, corresponding to coupling strength of 0.54 ~ 1.77 and 1.08 ~ 3.55, respectively. Then, both facets of these devices were HR coated by e-beam evaporation. The HR coating consisted of Al_2_O_3_/Ti/Au/Al_2_O_3_ (200/10/100/120). The devices were mounted epi-layer down on copper heat sinks with indium solder, and then, wire bonded to an external contact pad.

Device testing was done on a thermo-electric cooler (TEC) stage with a thermistor regulating and monitoring the temperature of the heat sink. The output power of the QCL was measured by a calibrated thermopile detector (Coherent, EMP1000) that was put right in front of the device with a metallic tube collecting the laser emission. Then, the device was put on a step-motor controlled rotational stage with a resolution of 0.01^o^ for far-field distribution test and a room temperature HgCdTe detector (Vigo, PVMI-10.6) was put in front of the laser with a distance of 30 cm to detect the radiation. The spectra measurement was carried out using a Fourier transform infrared (FTIR) spectrometer (Thermo Fisher Scientific, Nicolet 8700) with a resolution of 0.25 cm^− 1^ in rapid scan mode.

## Results and Discussion

The COMSOL simulation result is shown as Fig. 1b. According to the calculation, a coupling coefficient of |κ| = 35.5 ~ 10.75 cm^− 1^ is obtain for the buried grating with duty cycle of 0.35 ~ 0.45 and etching depth of 180 nm. The coupling strength *g* = |κ|*L*, where *L* is the cavity length of the QCL. To obtain efficient surface emission, the coupling strength of 1–2 is always required. For a device with 1 and 0.5 mm cavity-length, the simulated coupling strength are in the range of 3.55 ~ 1.07 and 1.78 ~ 0.54 when the duty cycle is 0.35 ~ 0.45. Therefore, the design of the buried grating is very essential for short cavity-length device.

**Fig. 2:**
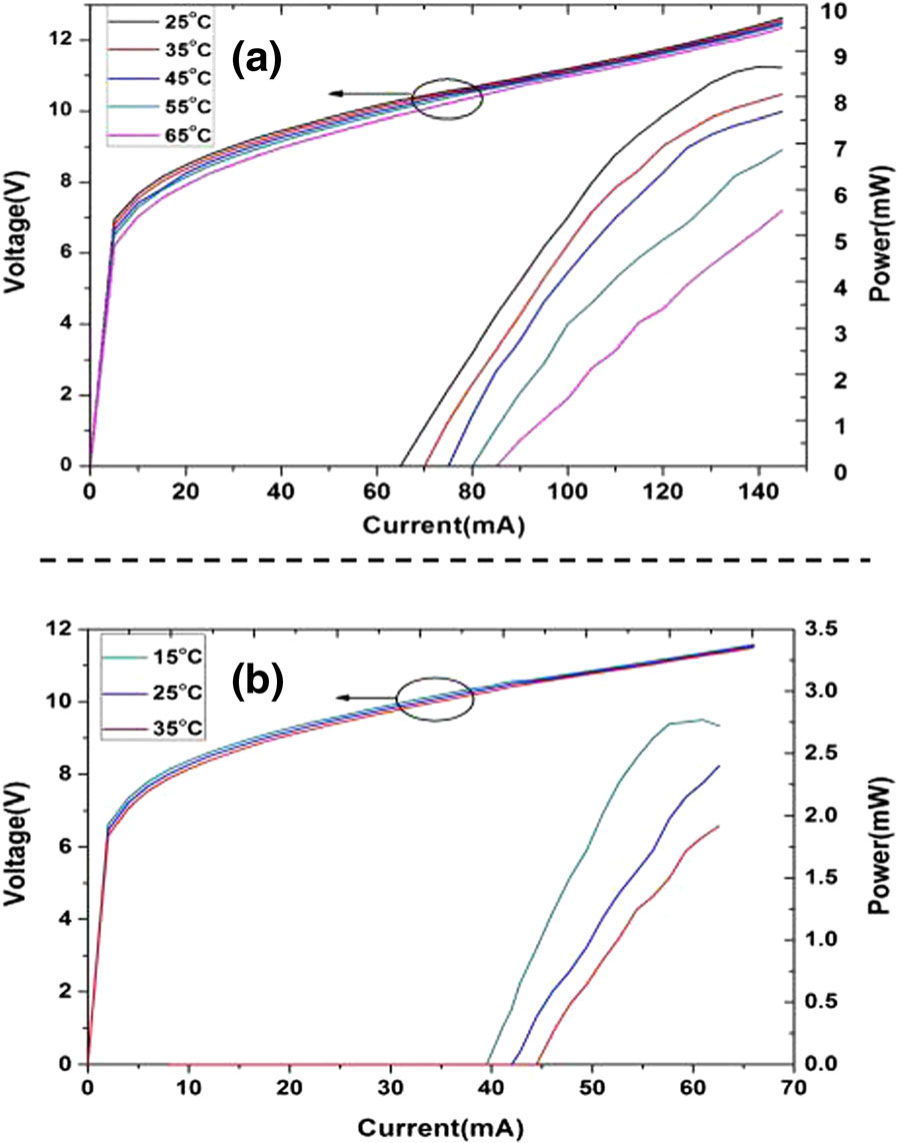
The L-I-V characteristics of the 1 mm (**a**) and 0.5 mm (**b**) devices

Of particular interest is the electrical characterization. The light-current-voltage (L-I-V**)** curve of the devices with different cavity length is shown as Fig. 2. The lasers operated in CW mode and the heat sink temperature was regulated by a temperature controller (Thorlabs, ITC-QCL-4000). As shown in Fig. 2a, the device with 1 mm-long cavity exhibits a threshold current of 65 mA at 25 °C, corresponding to a threshold current density of 0.54 kA/cm^− 2^ and power dissipation of 0.67 W. The maximum optical power is 8.6 mW with an injected electrical power of 1.66 W, and the slope efficiency is 0.11 W/A. At high temperature of 65 °C, the maximum optical power is still more than 5 mW. For a 0.5 mm cavity-length device, the threshold current and power dissipation are decreased to 39 mA and 0.4 W at 15 °C, as shown in Fig. 2b. The threshold current density is 0.65 kA/cm^− 2^. The maximum optical power of 2.8 mW is deduced when the injected electrical power is 0.74 W. At 25 °C, the threshold current is slightly increased to 41 mA, corresponding to a power consumption of 0.43 W. As far as we know, this is the lowest threshold power consumption of QCLs at the same temperature. The maximum optical power of this device is 2.4 mW with a power dissipation of 0.76 W, which is very capable of some high-integrated sensor applications. At 35 °C, the maximum optical power is about 1.9 mW. This device can operate at temperature as high as 105 °C in CW mode, but the output power will become small and too difficult to detect accurately. Compared to the previous works in Ref [9–11], the maximum optical power of our design is not remarkable because of the low wall-plug efficiency of the device. This is inherently limited by the quality of the epitaxy wafer. Besides, the maximum wall plug efficiency of 0.5 mm cavity-length device is 0.32% at room temperature, less than that of 1 mm cavity-length device, i.e., 0.5%.

**Fig. 3:**
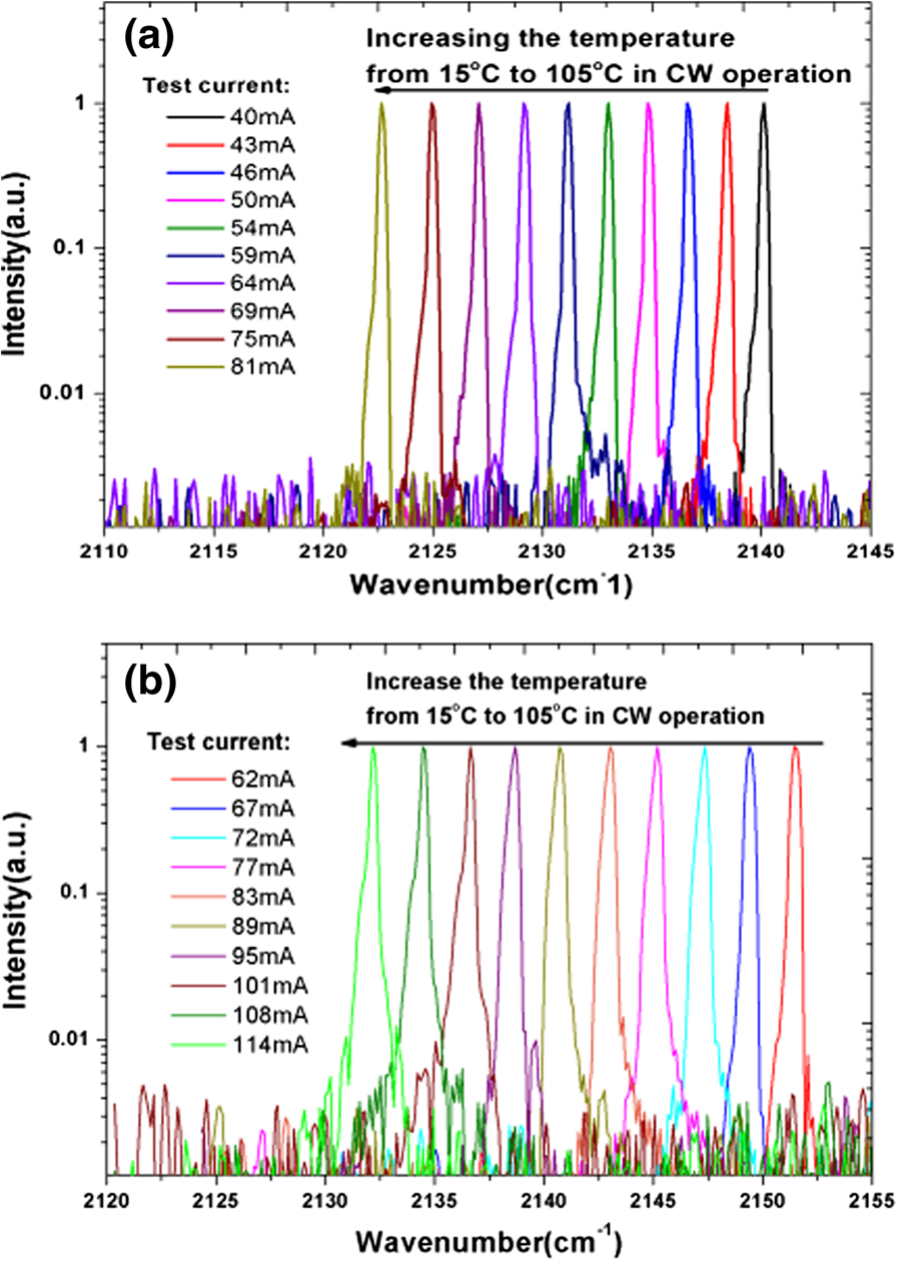
The lasing spectra of the **a** 0.5 and **b** 1 mm cavity-length device

The spectra characterization of the lasers is shown in Fig. 3. Both of the 1 and 0.5 mm devices can operate in CW mode without mode hop within a wide temperature range from 15 to 105 °C. This is the highest working temperature in all of the low power consumption QCLs. Such a high working temperature is mainly benefited from the reduced mirror loss brought by the HR coating on the facets. The temperature shift coefficient is − 0.21 cm^− 1^/K and − 0.19 cm^− 1^/K, respectively. There is a small difference between the spectra regimes of the two devices at the same temperature range, which is probably caused by the nonuniform lithography and etching process of the grating. The side-mode suppressing ratio (SMSR) of device is about 25 dB. The good linear tuning capability, single-mode and high operating temperature of these devices make them very useful in some real applications such as trace gas sensing.

**Fig. 4:**
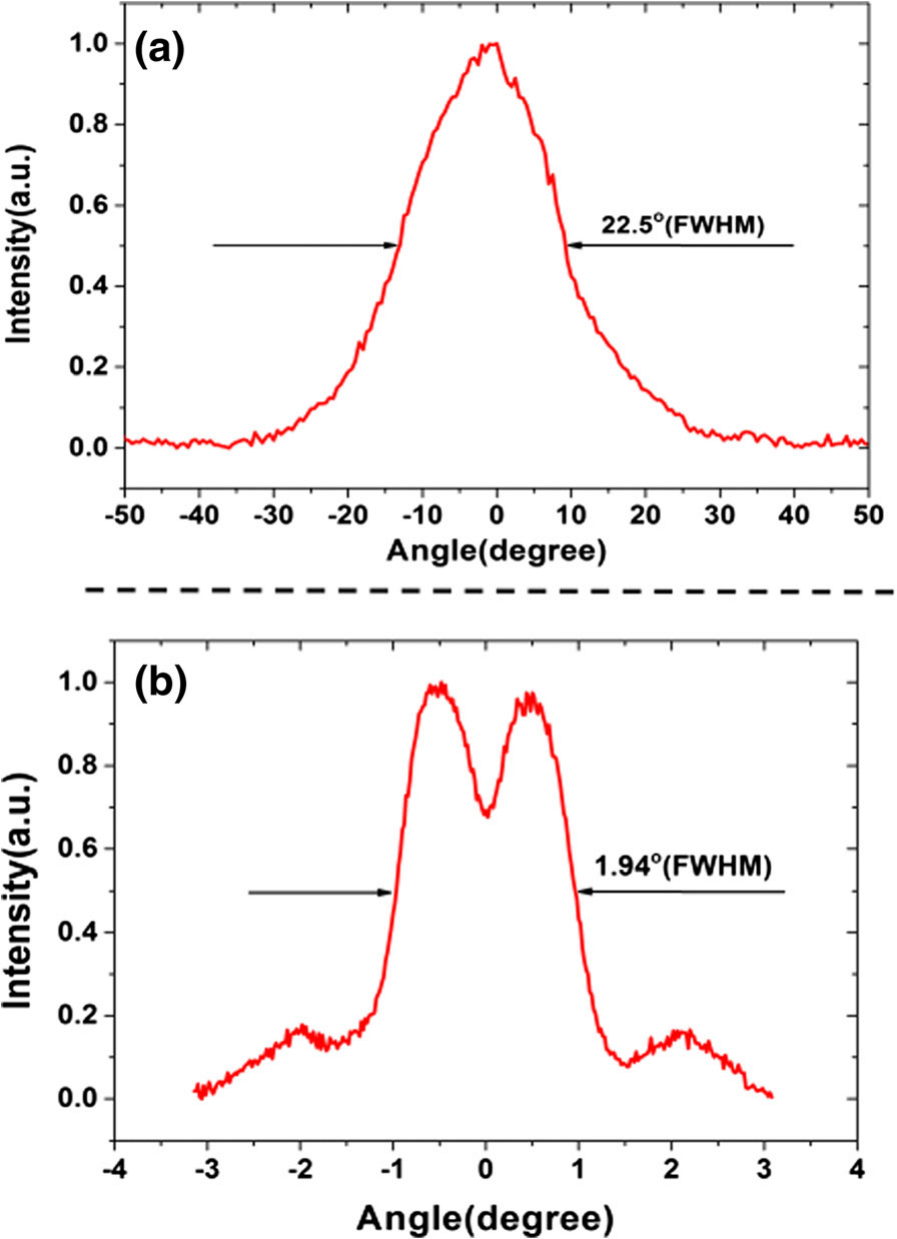
The far-field distribution of a 0.5-mm cavity-length device. **a**, **b** The far-field distributions in the ridge-width and cavity-length direction, respectively

The far-field distribution of a 0.5 mm device is shown in Fig. 4. In ridge-width direction, a single-lobed pattern with divergence angle of 22.5^o^ (FWHM) is observed, as shown in Fig. 4a. Figure 4b shows the far-field pattern in the cavity-length direction. The far-field pattern indicates that anti-symmetric mode is favored, which is caused by the nonuniformities of handmade cleaving and residual facet reflections [16]. Symmetric mode can be obtained by the use of distributed Bragg reflector (DBR) grating on both side of the DFB grating region to eliminate the uncontrolled cleaved facets reflections [17].

## Conclusions

We have developed a substrate-emitting DFB QCL with an ultra-low threshold power dissipation of 0.43 W at 25 °C operating in CW mode by shortening the cavity length to 0.5 mm and depositing HR coating on both of the facets. Its maximum optical power was 2.4 mW, and the corresponding power dissipation was 0.76 W. Single-mode emission without mode-hop was obtained within a wide temperature from 15 to 105 °C by defining a buried second-order DFB grating. The divergence angles are 22.5^o^ and 1.94^o^ in the ridge-width direction and cavity-length direction, respectively. The low-consumption characteristic of the device could make it a promising light source in some battery-powered portable systems.

## Abbreviations

CW: Continuous wave
DFB: Distributed feedback
FP: Fabry-Perot
FTIR: Fourier-transform infrared
FWHM: Full width at half maximum
HR: High reflectivity
MBE: Molecular beam epitaxy
MOVPE: Metal-organic vapor phase epitaxy
PECVD: Plasma-enhanced chemical vapor deposition
PHR: Partial high reflectivity
QCL: Quantum cascade laser
SEM: Scanning electron microscope
SMSR: Side-mode suppressing ratio
TEC: Thermo-electric cooler
